# Supplementary material for: Peptides from the Intestinal Tract of Breast Milk-Fed Infants Have Antimicrobial and Bifidogenic Activity
Source: Int J Mol Sci. 2021 Feb 27;22(5):2377. doi: 10.3390/ijms22052377 (PMC7956819; doi:10.3390/ijms22052377)
Supplement: Supplementary file 1 [file ijms-22-02377-s001.pdf]

Infant	GA[1]	Collection	Day of Life	Sample ID	Activity[2] *S. aureus*	Activity[2] *E. coli*	Activity[2] *B. infantis*
1	25/6	1	32	1	-	+	
		2	33	2	+	+	
2	29/4	1	50	3	+	+	
		2	50	4	-	+	
3	30/6	1	37	5	+		-
		2	37	6	-		
4	27/0	1	41	7	+		
		2	42	8	+		
		3	43	9	+	+	-
5	34/2	1	10	10	+	+	
		2	10	11	+		
6	33/1	1	7	12	+		+
		2	8	13	+		
7	31/0	1	25	14	+	+	
8	33/3	1	6	15	+		
		2	8	16	+		
9	36/3	1	33	17	+	+	
		2	34	18		+	
		3	34	19		+	+
10	37/4	1	57	20			
11	34/5	1	55	21	-	+	-
12	35/2	1	8	22	+	+	+
		2	8	23	+	+	
13	34/2	1	24	24	+	+	+
		2	24	25	+	+	-
14	34/3	1	7	26	+	+	+
15	39/2	1	27	27	+	+	+
		2	27	28	-	+	-
16	41/3	1	15	29	+	+	

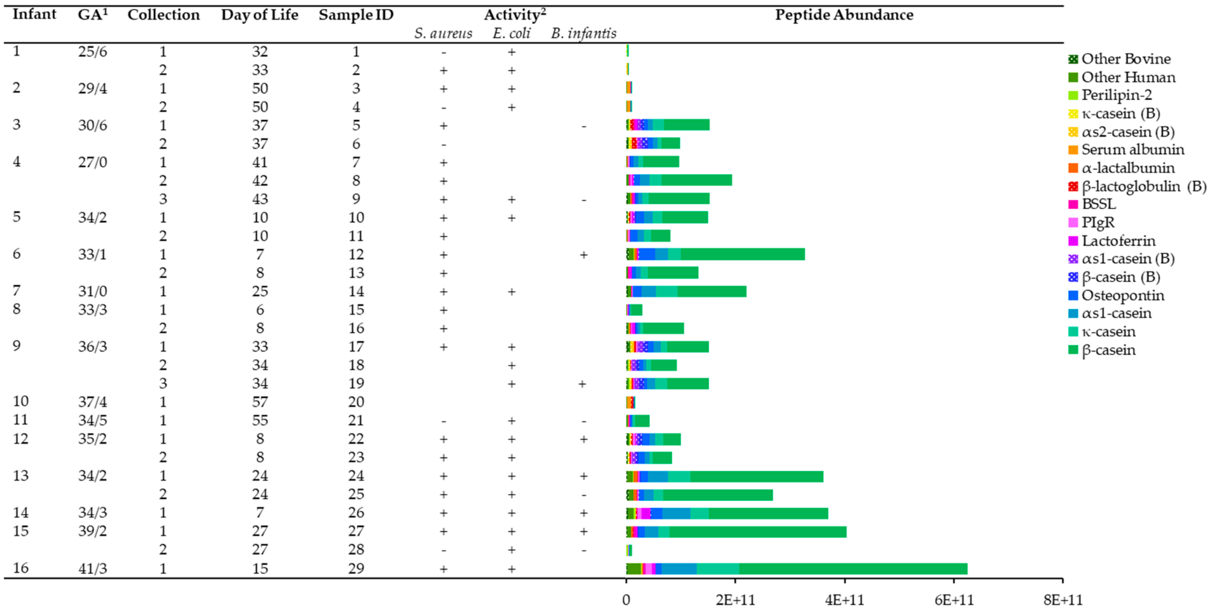

Figure S1. Infant birth information, screening activity, and total abundance of peptides from individual proteins for each intestinal peptide extract. Peptide abundances from each intestinal sample are sorted by protein from highest to lowest mean abundance. Bovine milk proteins are labeled with a (B) in the legend and are filled with a dotted pattern. PIgR, polymeric immunoglobulin receptor; BSSL, bile salt-stimulated lipase. [1] GA units are wk/day. [2] + Indicates the sample stimulated growth of the bacteria, - indicates the sample inhibited growth, and no symbol indicates the sample was inactive.

(**a**)

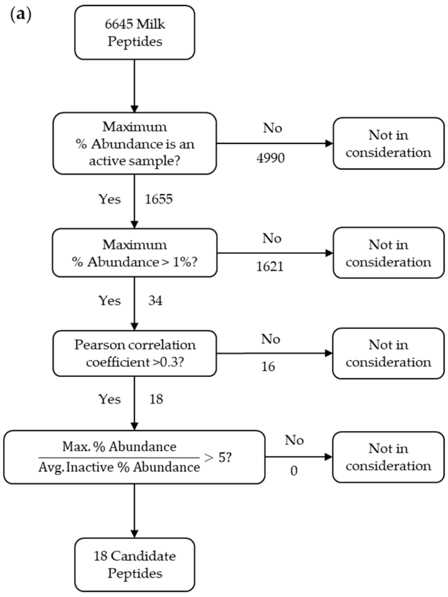

(**b**)

Sequence	Modifications	Protein	Start	Stop	Max % Abundance	Pearson Coefficient	Ratio	Sequence Homology
**MTSALPIIQK**		Perilipin-2	62	71	20.05	0.41	11515	
**HLPLPLLQPLMQQVPQPI**		β-casein	140	157	12.00	0.40	215	
EIENKAIQDPR		PIgR	593	603	11.74	0.41	451	
DLENLHLPL		β-casein	135	143	3.87	0.42	124	
DETYVPK		Serum albumin	518	524	3.63	0.40	23	
**FKDLGEENFK**		Serum albumin	35	44	2.61	0.54	18	
TVDGPSGKLW		GAPDH	187	196	2.38	0.42	870	
**EKFGKDKSPKFQ**		Lactoferrin	295	306	2.19	0.41	1673	
**RPNLHPS**		κ-casein	110	116	1.88	0.40	151	
QYKLGPKTGPGQ	Phospho [T8]	Fibroblast growth factor 2*	132	143	1.67	0.44	31	
**EPIPLESREE**		α_{s1}-casein	35	44	1.50	0.51	26	
RETIESLSSSEE		β-casein	16	27	1.41	0.42	19	
QLFGSPSGQK		Lactoferrin	306	315	1.27	0.40	26	
LPLRYPERLQNPSESSEPIPLESREEY		α_{s1}-casein	19	45	1.22	0.47	63	
ESSSHHPGIAEFPSRGK		Fibrinogen α-chain	559	575	1.17	0.40	340	
ADAAPDEKVLDSG		PIgR	578	590	1.16	0.42	66	
ILDIKGID		α-lactalbumin	114	121	1.16	0.32	26	
HQPHQPLP		β-casein*	160	167	1.13	0.40	115	

Figure S2. (a) Flow diagram of criteria and thresholds by which peptides with potential antimicrobial activity against *Staphylococcus aureus* were excluded from selection for activity testing. (b) List of peptides from which candidate peptides (in bold font) were selected for synthesis and activity testing.

(**a**)

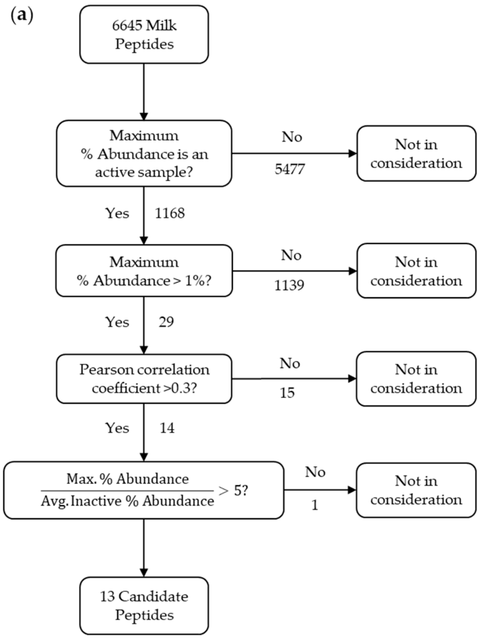

(**b**)

Sequence	Modifications	Protein	Start	Stop	Max % Abundance	Pearson Coefficient	Ratio	Sequence Homology
**LAPVHNPI**		β-casein	217	224	4.66	0.39	207	
LLNPTHQIYP		β-casein	203	212	3.44	0.32	20	
**HQIYPVTQPL**		β-casein	208	217	3.33	0.37	49	
**LLNPTHQIYPVTQPLAPVHNPIS**		β-casein	203	225	3.02	0.32	14	85.20
**YANPAVVRPHAQIPQR**		κ-casein	81	96	2.69	0.3	9	
VTQPLAPVHNP		β-casein	213	223	2.27	0.41	239	
LWSVPQPK		β-casein	168	175	2.06	0.35	18	
VTQPLAPVHNPI		β-casein	213	224	1.94	0.37	34	
LAPVHNPIS		β-casein	217	225	1.83	0.41	321	
**DMLVVDPK**		Osteopontin	283	290	1.34	0.36	41	
LLNPTHQIYPVTQPLAPVHNPI		β-casein	203	224	1.28	0.31	5	81.48
PVLKSPTIP		β-casein	116	124	1.14	0.33	9	
LMQQVPQPIPQT		β-casein	149	160	1.07	0.44	12	

Figure S3. (a) Flow diagram of criteria and thresholds by which peptides with potential bifidogenic activity for *Bifidobacterium infantis* were excluded from selection for activity testing. (b) List of peptides from which candidate peptides (in bold font) were selected for synthesis and activity testing.

Table S1. List of known bioactive peptides in the infant intestinal samples.

Peptide	Species	Parent Protein Location	Function
ENLHLPLP	Both	β-casein (137–144)	ACE-inhibitory
NLHLPLP	Both	β-casein (138–144)	ACE-inhibitory
ENLHLPLPLL	Both	β-casein (146–155)	ACE-inhibitory
NLHLPLPLL	Both	β-casein (147–155)	ACE-inhibitory
FFVAPFPEVFGK	Bovine	α_{s1}-casein (38–49)	ACE-inhibitory
FVAPFPEVFG	Bovine	α_{s1}-casein (39–48)	ACE-inhibitory
FPEVFGK	Bovine	α_{s1}-casein (43–49)	ACE-inhibitory
IGSENSEKTTMP	Bovine	α_{s1}-casein (201–212)	ACE-inhibitory
ALNEINQFYQK	Bovine	α_{s2}-casein (96–106)	ACE-inhibitory
AMKPWIQPK	Bovine	α_{s2}-casein (204–212)	ACE-inhibitory
LVYPFPGPI	Bovine	β-casein (73–81)	ACE-inhibitory
MPFPKYPVEP	Bovine	β-casein (124–133)	ACE-inhibitory
VENLHLPLPLL	Bovine	β-casein (145–155)	ACE-inhibitory
LLYQEPVLGPVRGPFPIIV	Bovine	β-casein (206–224)	ACE-inhibitory
QEPVLGPVRGPFPIIV	Bovine	β-casein (209–224)	ACE-inhibitory
EPVLGPVRGPFP	Bovine	β-casein (210–221)	ACE-inhibitory
VLGPVRGPFP	Bovine	β-casein (212–221)	ACE-inhibitory
LDIQKVAGTW	Bovine	β-lactoglobulin (28–37)	ACE-inhibitory
DAQSAPLRVY	Bovine	β-lactoglobulin (49–58)	ACE-inhibitory
FSDKIAK	Bovine	κ-casein (39–45)	ACE-inhibitory
HPHPHLSF	Bovine	κ-casein (119–126)	ACE-inhibitory
DKIYPSFQPQPLIYP	Human	β-casein (53–67)	ACE-inhibitory
IYPSFQPQPLIYP	Human	β-casein (55–67)	ACE-inhibitory
FQPQPLIYP	Human	β-casein (59–67)	ACE-inhibitory
TVYTKGRVMP	Human	β-casein (107–116)	ACE-inhibitory
LTDLENLHLP	Human	β-casein (133–142)	ACE-inhibitory
LENLHLPLP	Human	β-casein (136–144)	ACE-inhibitory
YANPAVVRP	Human	κ-casein (81–89)	ACE-inhibitory
IKHQGLPQEV	Bovine	α_{s1}-casein (21–30)	Antimicrobial
LRLKKYKVPQL	Bovine	α_{s1}-casein (114–124)	Antimicrobial
SDIPNPIGSENSEK	Bovine	α_{s1}-casein (195–208)	Antimicrobial
TKKTKLTEEEKNRL	Bovine	α_{s2}-casein (163–176)	Antimicrobial
IQPKTKVIPYVR	Bovine	α_{s2}-casein (209–220)	Antimicrobial
TKVIPYVRYL	Bovine	α_{s2}-casein (213–222)	Antimicrobial
TEDELQDKIHPF	Bovine	β-casein (56–67)	Antimicrobial
PVVVPPFLQPE	Bovine	β-casein (96–106)	Antimicrobial
VLPVPQKAVPYPQR	Bovine	β-casein (185–198)	Antimicrobial
YQEPVLGPVRGPFPI	Bovine	β-casein (206–220)	Antimicrobial
GLDIQKVAGT	Bovine	β-lactoglobulin (27–36)	Antimicrobial
AASDISLLDAQSAPLR	Bovine	β-lactoglobulin (41–56)	Antimicrobial

Table S1 (cont.)

Peptide	Species	Parent Protein Location	Function
IIAEKTKIPAVF	Bovine	β-lactoglobulin (89–100)	Antimicrobial
FSDKIAK	Bovine	κ-casein (39–45)	Antimicrobial
MAIPPKKNQDKTEIPTINT	Bovine	κ-casein (127–145)	Antimicrobial
LLNQELLLNPTHQIYPV	Human	β-casein (197–213)	Antimicrobial
QELLLNPTHQIYPVTQPLAPVHNPISV	Human	β-casein (200–226)	Antimicrobial
QVVPYPQ	Human	β-casein (182–188)	Antioxidant
YLGYLEQLLR	Bovine	αs1-casein (106–115)	Anxiolytic
ALKALPMHIR	Bovine	β-lactoglobulin (155–164)	Cell-proliferative
RETIESLSSSEESITEYK	Human	β-casein (16–33)	Cell-proliferative
SPTIPFFDPQIPK	Human	β-casein (120–132)	Cell-proliferative
LIVTQTMK	Bovine	β-lactoglobulin (17–24)	Cytotoxic
LPQNIPPLT	Bovine	β-casein (85–93)	DPP-IV-inhibitory
LKPTPEGDL	Bovine	β-lactoglobulin (62–70)	DPP-IV-inhibitory
LKPTPEGDLE	Bovine	β-lactoglobulin (62–71)	DPP-IV-inhibitory
INNQFLPYPY	Bovine	κ-casein (72–81)	DPP-IV-inhibitory
VYVEELKPTPEGDLEILLQK	Bovine	β-lactoglobulin (57–76)	Hypocholesterolemic
LYQEPVLGPVRGPFPIIV	Bovine	β-casein (207–224)	Immunomodulatory
VYPFPGPI	Bovine	β-casein (74–81)	PEP-inhibitory
ALPMHIR	Bovine	β-lactoglobulin (158–164)	ACE-inhibitory<br>Cell-proliferative
FALPQYLK	Bovine	αs2-casein (189–196)	ACE-inhibitory<br>Antioxidant
IQKVAGTW	Bovine	β-lactoglobulin (28–35)	ACE-inhibitory<br>DPP-IV-inhibitory
LKALPMH	Bovine	β-lactoglobulin (156–162)	ACE-inhibitory<br>DPP-IV-inhibitory
PYVRYL	Bovine	αs2-casein (217–222)	ACE-inhibitory<br>Antimicrobial<br>Antioxidant
RELEELNVPGEIVESLSSSEESITR	Bovine	β-casein (16–40)	Caseinophosphopeptide<br>Immunomodulatory
TPEVDDEALEK	Bovine	β-lactoglobulin (141–151)	Antimicrobial<br>DPP-IV-inhibitory
VKEAMAPK	Bovine	β-casein (113–120)	Antimicrobial<br>Antioxidant
VLVLDTDYK	Bovine	β-lactoglobulin (108–116)	Antimicrobial<br>DPP-IV-inhibitory
VYPFPGPIPN	Bovine	β-casein (74–83)	ACE-inhibitory<br>Antioxidant
WSVPQPK	Human	β-casein (169–175)	ACE-inhibitory<br>Antioxidant

Table S1 (cont.)

Peptide	Species	Parent Protein Location	Function
YQEPVLGPVR	Bovine	β-casein (208–217)	ACE-inhibitory<br>Immunomodulatory
YQEPVLGPVRGPFPIIV	Bovine	β-casein (208–224)	ACE-inhibitory<br>Antimicrobial<br>Antithrombin<br>Immunomodulatory
YQKFPQY	Bovine	αs2-casein (105–111)	ACE-inhibitory<br>Antioxidant
